# Proteomic Analysis of Rice Nonhost Resistance to *Puccinia striiformis* f. sp. *tritici* Using Two-Dimensional Electrophoresis

**DOI:** 10.3390/ijms151221644

**Published:** 2014-11-25

**Authors:** Jing Zhao, Yuheng Yang, Zhensheng Kang

**Affiliations:** State Key Laboratory of Crop Stress Biology for Arid Areas and College of Plant Protection, Northwest A&F University, No. 3 Taicheng Road, Yangling 712100, China; E-Mails: zhaojing@nwsuaf.edu.cn (J.Z.); yyh023@swu.edu.cn (Y.Y.)

**Keywords:** nonhost resistance, two-dimensional polyacrylamide gelelectrophoresis (2D-PAGE), proteomics, wheat stripe rust

## Abstract

Rice (*Oryza sativa* L.) is the only widely cultivated gramineous crops that cannot be infected by rust fungi. To decipher the molecular basis of rice nonhost resistance (NHR) to *Puccinia striiformis* f. sp. *tritici* (*Pst*), the causal agent of wheat stripe rust*,* proteomic analysis was performed using the two-dimensional electrophoresis (2-DE) technique. The expressed proteins from rice leaves 24 and 48 h post inoculation with *Pst* and from mock-inoculated leaves were identified. Quantitative analysis revealed a total of 27 differentially expressed proteins in response to *Pst* inoculation. Most of these proteins fall into the category “response to stimulus” and are involved in basic resistance processes, such as glycerol-3-phosphate and hydrogen peroxide signaling. A homologue of wheat leaf rust resistance protein Lr10 was also identified, implicating multiple layers of plant defense are implicated in rice NHR to *Pst*. These results demonstrate an intrinsic relationship between host and nonhost resistance. Changes in abundance of these proteins, together with their putative functions reveal a comprehensive profile of rice NHR to *Pst* and provide new insights into plant immunity.

## 1. Introduction

Wheat stripe rust, caused by *Puccina striiformis* f. sp. *tritici* (*Pst*), is one of the most devastating fungal diseases of wheat, and leads to significant yield losses worldwide [1]. Due to the frequent virulence variation of the pathogen, several resistance genes have been defeated by new races [2]. Nonhost resistance (NHR) provides immunity to all members of a plant species against all isolates of a pathogen species that normally infects other plant species [3,4]. As the most common form of plant resistance, NHR is remarkable for its broad-spectrum effectiveness and durability, and has therefore attracted much attention owing to its potential for improving crop resistance [3,5,6].

In recent years, the interactions between non host plants and rust fungi have been studied at histocytological and genetic levels [7,8,9,10]. In most cases, plant responses to non-adapted rust fungi were similar to the basic defense responses to the adapted rust fungi. For example, interaction between *Arabidopsis* and non-adapted *Puccinia triticina* (*Ptr*, wheat leaf rust fungus) induces production of nitric oxide (NO), salicylic acid (SA), camalexin and transient stomatal closure [9]. Genetic studies revealed that multiple quantitative trait loci (QTL) and occasionally resistance genes (*R*) conferred NHR in barley to heterologous rust species. Moreover, most of these genes were effective against only one non-adapted rust species [11,12]. These findings reveal that multiple genetic and molecular components contribute to NHR, indicating that a multitude of underlying mechanisms determine the outcome of diverse nonhost interactions.

As a model plant for monocotyledonous crops, rice (*Oryza sativa* L.) is a nonhost species to all known rust fungi. Histocytological studies demonstrated that multiple defense responses, including callose deposition, production of reactive oxygen species and cell death, contributed to rice NHR against several rust species. Rice mutants lacking some defense-related or basic defense genes, did not exhibit increased susceptibility to rust fungi [13]. These results suggested that the mechanisms underlying rice NHR were different from basic resistance despite the similar responses.

Proteomic approaches have been extensively applied in plant pathology research [14]. However, only a few studies have examined changes in the plant proteome in response to non-adapted pathogens, especially to rust pathogens. To understand the molecular basis of rice NHR to rust fungi, we identified a set of NHR-related proteins in rice plants that were inoculated with *Pst* using two-dimensional electrophoresis (2-DE). Results are discussed according to the functional implications of the proteins identified, with special emphasis on their possible roles in defense response. This study extends our knowledge on NHR and allows us to further understand plant immune systems.

## 2. Results

### 2.1. Rice Proteome Induced by Pst Infection

The loading quantities of protein samples have significant impact on the results of 2-DE. In a preliminary study 400, 500 and 600 μg of protein extracts were used to optimize the quantity of the loading samples. A loading sample of 500 μg generated well-resolved gel maps and was selected as the standard for subsequent analysis (data not shown). 

**Figure 1:**
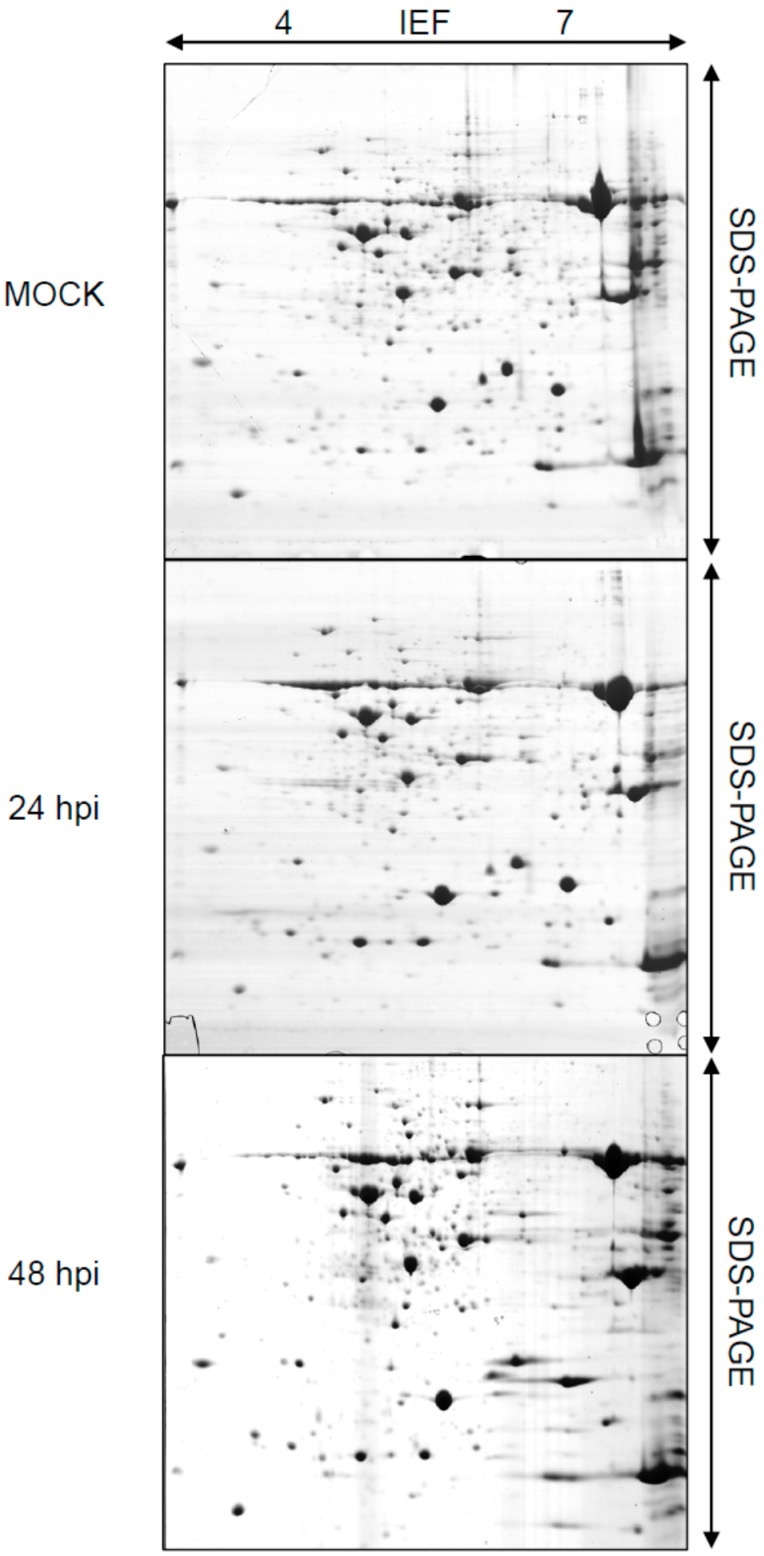
Two-dimensional electrophoresis (2-DE) gel maps with proteins isolated from rice leaves inoculated with water (MOCK) or *Puccina striiformis* f. sp. *tritici* (*Pst*) (harvested at 24 and 48 h post inoculation (hpi)). Proteins were first separated by their pI through isoelectric focusing (IEF) and then further separated by molecular weight through SDS-PAGE.

**Figure 2:**
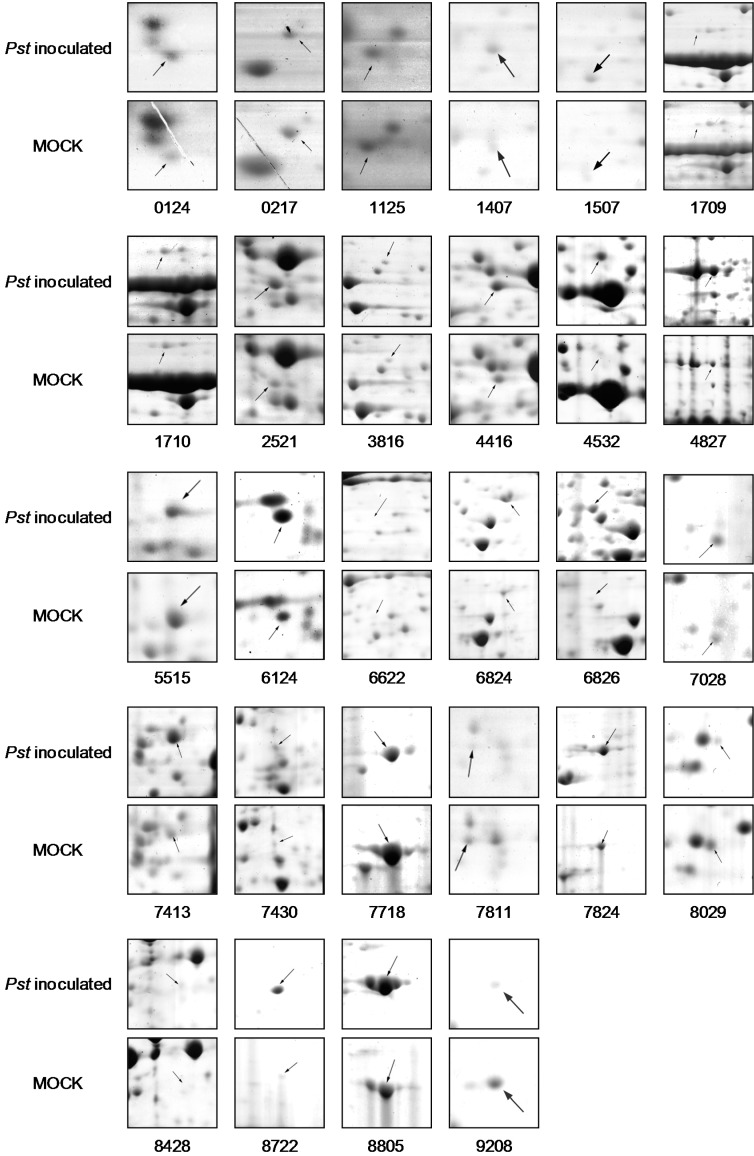
Differentially expressed proteins induced by *Pst*. Arrows point to protein spots with altered expression levels (fold change > 2.0, *p* < 0.05).

To ensure successful inoculation, leaf samples from 24 and 48 h post inoculation (hpi) treatments were collected and subjected to histological examination (Supplementary Figure S1). Rice leaf proteins from 24 and 48 hpi treatments or mock controls were analyzed by 2-DE. A total of 878 spots from each sample were resolved (Figure 1). Comparative analysis revealed 30 spots showing significant differences (fold change > 2.0) in protein abundance between inoculated and control plants. Among them, 15 spots were obtained from samples at 24 and 48 hpi respectively (Table 1 and Table 2; Figure 2).

**Table 1:** Differentially expressed proteins in rice leaves at 24 h post inoculation with *Puccinia striiformis* f. sp. *tritici*.

Spot No.	Identification	GenBank Accession No.	pI/MW (kDa) ^a^	Peptides Count ^b^	Coverage (%) ^c^	Protein Score/Ion Score	Mock Average Qty	Inoculated Average Qty	Fold Change
**Up-Regulated Expression**									
124	Ribulosebiphosphate carboxylase	gi|224612718	5.96/21.5	6	34.7	95/143	307.4 ± 3.1	1180.6 ± 19.3	3.84 ± 0.04
1125	Ribosomal protein S6	gi|125588601	5.97/23.1	5	29.8	138/171	444.5 ± 0.5	1118.3 ± 93.7	2.51 ± 0.21
1407	Phosphoglycerate kinase	gi|125552851	6.86/30.5	9	43.3	568/634	55.5 ± 9.0	572.5 ± 25.2	10.59 ± 1.71
1507	Chloroplast phosphoglycerate kinase	gi|46981258	9.93/32.5	10	38.4	229/300	0	238.9 ± 9.8	−
1709	OsAPx8, Thylakoid-bound ascorbateperoxidase	gi|125582491	4.79/43.6	12	35.7	250/333	0	176.8 ± 3.9	−
1710	OsAPx8, Thylakoid-bound ascorbateperoxidase	gi|115446663	5.36/51.4	21	44.8	348/538	78.4 ± 0.4	424.2 ± 0.4	5.41 ± 0.01
2521	Glyceraldehyde-3-phosphate dehydrogenase b	gi|115450493	6.22/47.5	19	48.4	648/810	355.2 ± 7.4	1333.9 ± 4.5	3.76 ± 0.08
3816	OsFtsH1, FtsH protease	gi|115470052	5.87/67.0	15	24.5	149/247	52.4 ± 3.7	122.9 ± 10.1	2.34 ± 0.01
4416	Glyceraldehyde-3-phosphate dehydrogenase	gi|115458768	7.62/43.0	18	54.2	864/1030	660.8 ± 23.2	1636.4 ± 31.3	2.48 ± 0.09
7122	Ribulose-bisphosphate carboxylase oxygenase large subunit	gi|297720471	6.35/29.1	14	39.7	408/621	1607.2 ± 32.7	8024.3 ± 34.2	5.22 ± 1.08
7413	Glyceraldehyde-3-phosphate dehydrogenase	gi|115458768	7.62/43.0	21	59.2	716/923	342.25 ± 11.9	1922.6 ± 31.3	6.16 ± 1.83
0217	ATP synthase F1, delta subunit family protein	gi|115448701	4.98/26.2	8	31.7	378/439	739.1 ± 3.1	30.3 ± 0.3	0.04 ± 0.04
5515	Aminotransferase	gi|115477483	6.48/50.4	17	46.9	704/847	916.1 ± 31.5	282.5 ± 134.8	0.31 ± 0.15
**Down-Regulated Expression**									
6622	NAD dependent epimerase/dehydratase family protein	gi|115482032	5.75/43.2	23	65.9	418/635	955.9 ± 47.3	353.9 ± 103.9	0.37 ± 0.11
7811	Ferredoxin-nitrite reductase	gi|297599961	6.63/47.1	5	13.4	66/94	122.3 ± 14.9	52.2 ± 1.6	0.43 ± 0.01

a: pI of predicted protein/molecular mass of predicted protein (kDa); b: Number of peptides for protein identification; c: Percentage of protein sequence represented by peptides identified in MS.

**Table 2:** Differentially expressed proteins in rice leaves at 48 h post inoculation with *Puccinia striiformis* f. sp. *tritici*.

Spot No.	Identification	GenBank Accession No.	pI/MW (kDa) ^a^	Peptides Count ^b^	Coverage (%) ^c^	Protein Score/Ion Score	Mock Average Qty	Inoculated Average Qty	Fold Change
**Up-Regulated Expression**									
4532	Glyceraldehyde-3-phosphate dehydrogenase b	gi|115450493	6.22/47.5	14	31.8	641/747	359.7 ± 13.7	1349.8 ± 37.4	3.76 ± 0.14
4827	Transketolase, chloroplast precursor	gi|115466224	5.44/74.0	25	53.1	543/773	497.3 ± 23.6	1267.7 ± 35.4	2.55 ± 0.10
5318	Chloroplast ATP synthase CF1 alpha chain	gi|20143564	5.27/29.4	3	23.0	150/166	324.5 ± 10.6	732.9 ± 21.7	2.26 ± 0.10
6124	Ribulose-1,5-bisphophate carboxylase oxygenase	gi|354618517	5.80/19.7	8	38.2	268/340	729.0 ± 17.0	1702.1 ± 31.2	2.34 ± 0.08
6824	DnaK family protein, chloroplast Hsp70	gi|125578088	5.33/69.8	23	37.3	586/763	344.4 ± 24.8	1192.8 ± 84.6	3.48 ± 0.25
6826	OsFtsH1, FtsH protease	gi|115470052	5.51/72.9	13	25.8	587/668	44.2 ± 2.7	335.6 ± 21.2	7.59 ± 0.68
7028	Carbonic anhydrase	gi|5917783	8.41/29.6	7	41.8	203/253	451.3 ± 37.2	1196.4 ± 79.5	2.65 ± 0.25
7430	Chloroplast 28 kDa ribonucleo protein	gi|149391365	5.03/21.0	12	75.3	234/351	316.9 ± 0.3	1552.1 ± 13.0	4.90 ± 0.06
7824	Putative LR10 resistance protein	gi|305691143	6.08/105.4	8	14.7	13/37	284.7 ± 3.5	763.3 ± 17.7	2.68 ± 0.13
8428	Phosphoribulo kinase	gi|115448091	5.68/45.2	12	25.6	153/230	210.4 ± 1.3	432.7 ± 57.8	2.05 ± 0.26
8722	Rhodanese-like domain-containing protein chloroplastic-like	gi|115445387	5.05/48.4	6	15.5	215/238	108.0 ± 1.8	650.0 ± 15.9	6.03 ± 0.35
8805	Chloroplast Hsp70	gi|15233779	5.07/76.6	16	26.9	789/887	703.9 ± 13.5	2818.1 ± 105.0	4.00 ± 0.22
**Down-Regulated Expression**									
7718	T-complex protein	gi|115488160	5.12/61.1	23	38.6	683/882	2229.3 ± 60.5	859.0 ± 35.2	0.39 ± 0.01
8029	Thioredoxin	gi|297728925	8.16/18.9	3	23.8	82/99	897.8 ± 22.2	275.5 ± 7.4	0.31 ± 0.01
9208	Ribonucleoprotein chloroplastic-like	gi|149392545	4.45/22.2	6	35.1	420/462	1228.8 ± 35.4	403.5 ± 5.8	0.33 ± 0.01

a: pI of predicted protein/molecular mass of predicted protein (kDa); b: Number of peptides for protein identification; c: Percentage of protein sequence represented by peptides identified in MS.

### 2.2. Identification of Differentially Expressed Proteins by Mass Spectrometry

All differentially expressed spots were subjected to mass spectrometry analysis and identification (Table 1 and Table 2). The 30 differentially expressed spots represented 27 annotated proteins. Three proteins each identified by two spots included two glyceraldehyde-3-phosphate dehydrogenases (gi|115458768 and gi|115450493) represented by spots 4416/7413 and 2521/4532, and one FtsH protease (gi|115470052) represented by spots 3816/6826. This was probably due to posttranslational protein modifications or presence of homologous proteins. Ten proteins were up-regulated and four proteins were down-regulated at 24 hpi; 12 were up-regulated and three were down-regulated at 48 hpi. Two proteins, a glyceraldehyde-3-phosphate dehydrogenase (GAPDH, gi|115450493) and an FtsH protease (gi|115470052), were up-regulated at both time points.

**Figure 3:**
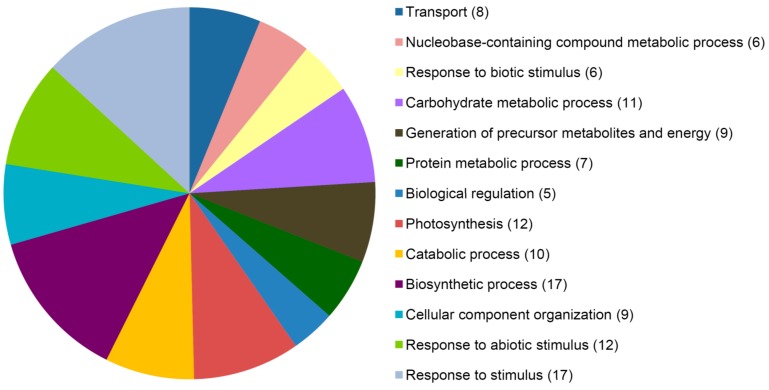
GO (gene ontology) Slim analysis of differentially expressed proteins in rice inoculated with *Pst*. Number of proteins attributed to each GO term are in parentheses.

All 27 proteins identified were analyzed for gene ontology using GO (gene ontology) Slim and classified by biological processes. As expected, 19 differentially expressed proteins were attributed to the class “response to stimulus” (Figure 3; Supplementary Table S1), accounting for 63.0% of all identified proteins. They were categorized according to function into six groups including energy metabolism, protein synthesis and modification, photosynthesis, glycerol-3-phosphate metabolism, oxidation-reduction processes and resistance protein. Four proteins, including one ATP synthase F1, delta subunit family protein (spot no. 0217), one chloroplast ATP synthase CF1 alpha chain protein (spot no. 5318), one rhodanese-like domain-containing protein (spot no. 8722) and one transketolase (spot no. 4827) were involved in energy metabolism. Four proteins associated with protein synthesis and modification included two Hsp70 family proteins (spot no. 6824 and 8805), one amino transferase (spot no. 5515) and one ribonucleo protein (spot no. 9208). Three proteins in the class of oxidation-reduction processes included OsAPx8 (spot no. 1710), one thylakoid-bound ascorbate peroxidase (spot no. 1709) and one thioredoxin (spot no. 8029). Three proteins involved in glycerol-3-phosphate metabolism included two phosphoglycerate kinase (spot no. 1407 and 1507) and one GAPDH (spot no. 2521/4532). Two proteins involved in photosynthesis included one FtsH protease (spot no. 3816/6826) and one phosphoribulokinase (spot no. 8428). A homologue protein of wheat Lr10, a host resistance protein that confers resistance to the wheat leaf rust fungus, was also identified. These results indicated that proteins involved in host resistance also have roles in the nonhost response.

### 2.3. Validation of Up-Regulated Proteins by qRT-PCR

To further confirm the changes in protein abundance, qRT-PCR was used to analyze the expression patterns of coding genes after inoculation. Fifteen up-regulated proteins were selected. The mRNA levels of 14 proteins exhibited significant increases (fold change > 2) in at least one sampling time (Table 3). The changes were consistent with the induced increases of corresponding proteins and validated the differentially expressed proteins identified by two-dimensional polyacrylamide gelelectrophoresis (2D-PAGE). Additionally, the mRNA levels of five proteins, including an Hsp70 (gi|125578088), a phosphoribulokinase (gi|115448091), a rhodanese-like domain-containing protein (gi|115445387), a transketolase (gi|115466224) and an ATP synthase CF1 alpha chain protein (gi|20143564), were induced as early as 12 hpi. The single transcript that remained stable over both time courses was the coding gene for FtsH protease, which was up-regulated at both 24 and 48 hpi. Since FtsH protease interacts with molecular chaperone Hsp70, we suggest it is stabilized by Hsp70 and therefore has an increased half-life, allowing it to persist in abundance at a relatively steady transcriptional level [15,16].

**Table 3:** Transcriptional changes of differentially expressed proteins induced by *Pst* relative to the mock control.

Accession No.	Gene	12 hpi	24 hpi	48 hpi	72 hpi
gi|125588601	*Os03g62630*	0.94 ± 0.32	2.65 ± 1.16	2.86 ± 0.84	1.25 ± 0.16
gi|46981258	*Os05g41640*	0.96 ± 0.19	2.06 ± 0.27	3.60 ± 1.18	0.85 ± 0.40
gi|125582491	*Os02g34810*	0.96 ± 0.14	2.31 ± 0.43	2.12 ± 0.75	0.87 ± 0.20
gi|115450493	*Os03g03720*	1.09 ± 0.05	3.77 ± 0.33	4.23 ± 0.41	1.91 ± 1.26
gi|115470052	*Os06g51029*	1.03 ± 0.16	0.68 ± 0.05	1.15 ± 0.29	0.63 ± 0.22
gi|115458768	*Os04g38600*	0.79 ± 0.50	3.41 ± 0.40	6.00 ± 2.13	1.11 ± 0.18
gi|115466224	*Os06g04270*	2.86 ± 0.74	2.86 ± 0.74	1.07 ± 0.31	0.51 ± 0.11
gi|20143564	*OSJNBa0034L04.44*	2.19 ± 0.70	0.89 ± 0.02	2.43 ± 0.68	2.30 ± 0.41
gi|5917783	*Os01g45274*	1.15 ± 0.23	0.88 ± 0.13	13.39 ± 0.54	0.84 ± 0.24
gi|149391365	*Os09g39180*	0.63 ± 0.19	0.83 ± 0.27	4.67 ± 0.64	1.84 ± 0.64
gi|115448091	*Os02g47020*	7.54 ± 0.85	2.91 ± 0.90	1.81 ± 0.48	1.13 ± 0.27
gi|115445387	*Os02g15750*	2.91 ± 1.70	0.70 ± 0.12	4.65 ± 0.74	0.99 ± 0.28
gi|15233779	*Os12g14070*	0.77 ± 0.09	1.35 ± 0.16	3.09 ± 0.19	1.33 ± 0.07
gi|305691143	*Os11g14380*	0.64 ± 0.09	1.84 ± 0.12	2.17 ± 0.19	2.39 ± 0.55
gi|125578088	*Os11g47760*	36.82 ± 9.84	12.18 ± 5.57	0.69 ± 0.21	1.20 ± 0.31

hpi: hours post inoculation.

## 3. Discussion

Our previous work indicated that the nonhost response of rice to *Pst* started at 24 hpi and was characterized by increasing hydrogen peroxide (H_2_O_2_) accumulation [17]. However, the time points selected in the previous proteomic studies were too late. For example, Liang *et al.* [18] identified wheat proteins 14 days after inoculation with *Pst*; Ma *et al.* [19] studied the wheat proteome 48 hpi with *Pst*; Li *et al.* [20] analyzed the rice proteins 3 days after inoculation with the leaf rust fungus. The relatively earlier time points (24 and 48 hpi) we chose may not only help us to find more relevant proteins, but also provide more accurate information regarding proteome reprogramming in rice at the early stages of *Pst* infection.

As shown in the results, GAPDH, a key enzyme in glycerol-3-phosphate metabolism was up-regulated at both 24 and 48 hpi. Another key enzyme linked to glycerol-3-phosphate metabolism, phosphoglycerate kinase (PGK), that acts downstream of GAPDH, was also induced and exhibited significant changes in abundance (10.6-fold higher than the control) at 24 hpi. GAPDH was also induced in disease-resistance enhanced rice mutants *spl5*, *spl1* and *cdr2* [21,22,23]. GAPDH mainly converts glyceraldehyde-3-phosphate into 1,3-bisphosphoglycerate, which is then converted to glycerol-3-phosphate by phosphoglycerate kinase in the glycolytic pathway [24]. Glycerol-3-phosphate is an important signal molecule in systemic acquired resistance in *Arabidopsis* and wheat [25,26]. A recent study indicated that the *Arabidopsis* GAPDH interacts with phospholipase Dδ on the plasma membrane, and is involved in abscisic acid (ABA) and H_2_O_2_ signal transduction, triggering stress responses such as stomatal closing [27]. It was also reported that PGK was involved in the hypoxic responses of rice and wheat [28]. These results showed that the GAPDH and PGK pathway plays an important role in the the nonhost response in rice. 

Numerous studies have demonstrated that heat shock protein Hsp70 plays an important role in the plant defense response. Hsp70-silenced tobacco exhibited a compromised HR response to *Phytophthora infestans* and impaired nonhost resistance to *Pseudomonas cichorii* [28]. Hsp70 and another heat shock protein, Hsp90, together with RAR1 and Rac1, two key components of the plant immune system, were found to form a complex, which may be critical in rice innate immunity [29]. In the present study, two Hsp70 proteins were up-regulated, indicating that both of them played a role in nonhost resistance. In rice, Hsp70 suppresses H_2_O_2_-induced programmed cell death [30]. We presumethat as a molecular chaperon, Hsp70 may protect key components of the rice immune system from degradation induced by H_2_O_2_ at the early stage of the nonhost defense response. This was supported by the accumulation of two FtsH proteases following *Pst* infection in the present study. Hsp70 protects precursors of FtsH2 and FtsH1 from degrading by 26S proteasome ubiquitylation, leading to H_2_O_2_ excess and HR responses under high-intensity light conditions [15]. H_2_O_2_ production occurred not only in infected mesophyll cells and stomatal guard cells, but also at attempted infection sites [13]. In a previous study, we found that compared to japonica rice plants, indica plants were more vulnerable to the infection by *Pst* due to lower H_2_O_2_ production, suggesting a critical role of H_2_O_2_ in rice NHR to *Pst* [17]. The expression levels of a major ROS detoxification enzyme, thylakoid ascorbate peroxidase OsAPx8 (Os02g34810) in inoculated plants was 5-fold higher at 24 hpi than that in the non-inoculated control, indicating that the reactive oxygen scavenging system was also involved in response to infection by the non-adapted pathogen. These results further demonstrate the complex regulation of H_2_O_2_ in the early stages of NHR.

Lr10, a typical CC-NBS-LRR (coiled-coil, nucleotide-binding site and leucine-rich repeat) type disease resistance protein in wheat, confers resistance to leaf rust fungus. Although it is encoded by a single-copy gene located on wheat chromosome 1AS, there are six homologous proteins in rice [31]. Among them, Os11g14380 was induced at 48 hpi in the presentstudy and another Lr10 homologue (Os08g29809) was also up-regulated in rice challenged by the leaf rust fungus [20]. Although NHR is mainly controlled by multiple quantitative loci, *R* genes have also been implicated in NHR. A typical R protein from maize, RXO1, confers resistance to *Xanthomonas oryzae* Pv. *oryzkola*, which causes bacterial leaf streak in rice [32]. Genetic studies demonstrated that NHR in wheat and barley crown rust (*Puccinia coronata* var. *hordei*) was controlled through one or two dominant genes in wheat accessions Chris and Chinese Spring, respectively [33]. Niks *et al.* [12] proposed that nonhost plants, especially near-nonhost, possess multiple *R* genes corresponding to *Avr* genes in potential pathogens. The possible role of rice Lr10 homologue in defense responses to rust fungi further supports participation of *R* genes in NHR.

Our proteomic study provided a macroscopic profile of rice nonhost response to *Pst* at the early stage of infection. Most of the differentially expressed proteins identified were known and commonly involved in host plant stress responses, such as H_2_O_2_ production and glycerol-3-phosphate signaling. Nonhost resistance may overlap with host basal resistance, and only a few key components may be specific for NHR. Since these key components are usually located upstream in the signaling pathway and are stable at the transcriptional level, mutant screening and genetic studies would be helpful to identify them in future studies.

## 4. Experimental Section

### 4.1. Plants and Pathogens

Plants of japonica rice (*Oryza sativa* L. ssp. *japonica*) cultivar Nipponbare were grown in a greenhouse at 25 °C with 16 h of light and at 20 °C in 8 h of darkness. *Pst* isolate CYR32 (a predominant *Pst* race in China from 2002) was maintained on the susceptible wheat line, Mingxian 169, following the procedures and conditions described by Zhang *et al.* [34]. Fresh aqueous *Pst* suspensions of urediniospores were applied with a fine paintbrush onto the adaxial surface of the second leaves of rice seedlings at the two-leaf stage. The inoculated seedlings were kept in a dew chamber in darkness for 36 h at 12 °C and then transferred to a growth chamber at 16 °C with a 16/8 h photoperiod. To ensure successful inoculation, leaf samples were collected and subjected to histological examination according to the method previously described [17]. Leaf tissues for various analyses were collected at specific time points post-inoculation. Controls inoculated with water were treated in the same way.

### 4.2. Protein Extraction

Harvested leaf tissues were ground to fine powder with liquid nitrogen. Total protein extraction was performed according to the trichloroacetic acid (TCA)-acetone precipitation method [35]. Protein concentrations were estimated using Bradford’s reagent (Sigma–Aldrich, St. Louis, MO, USA) [36].

### 4.3. Two-Dimensional Electrophoresis

For 2-DE, 500 μg of protein was loaded onto 17 cm isoelectric focusing (IEF) strips with pH 4 to 7 linear gradient (Bio-Rad, Richmond, CA, USA) according to the manufacturer’s protocol. The IEF conditions were: 50 V for 14 h, 250 V for 1 h, 500 V for 1 h, 1000 V for 1 h, 1000–8500 V for 5 h over a linear gradient, 8500 V for 6.5 h, and a 500 V hold. For the second dimension, the strips were first incubated in equilibration buffer I containing 2% dithiothreitol for 15 min and then replaced with equilibration buffer II containing 2.5% iodoacetamide for 15 min. After equilibration, proteins were separated by 12% SDS–PAGE and sealed with 1% agarose. Electrophoresis was carried out at 5 mA per gel for 1 h, and then 25 mA per gel for 6 h using a PROTEAN II XL machine (Bio-Rad). After electrophoresis, the gels were stained with Coomassie Brilliant Blue R-250 (Colab Laboratories Inc., Chicago, IL, USA). Two gels were run for each time point.

### 4.4. Image Analysis

Stained gels were scanned with a UMAX PowerLook Scanner (Bio-Rad). Protein spots were then detected, normalized, and quantified by PDQuest 8.0 (Bio-Rad). Proteins were considered significantly different between treated samples and controls when spot intensities passed a threshold of at least a two-fold difference in up- or down-regulation in combination with a Student’s *t*-test on concentrations using a 95% reliability score.

### 4.5. Mass Spectrometric Analysis and Protein Identification

Protein spots with at least two-fold differences (*p* < 0.05) were collected from the 2-DE gels for inoculated and control groups. Protein samples for MS were excised manually from the gels and prepared according to Shevchenko *et al.* [37]. MS and MS/MS data for protein identification were obtained by using a MALDI-TOF-TOF instrument (4800 proteomics analyzer; Applied Biosystems, Foster City, CA, USA). Instrument parameters were set using the 4000 Series Explorer software (Applied Biosystems). The MS spectra were recorded in reflector mode in a mass range from 800 to 4000 with a focus mass of 2000. A CalMix5 standard was used to calibrate the instrument (ABI 4700 calibration mixture; Applied Biosystems). For one main MS spectrum, 25 subspectra with 125 shots per subspectrum were accumulated using a random search pattern. For MS calibration, autolysis peaks of trypsin ((M + H) + 842.5100 and 2211.1046) were used as internal calibrates, and up to 10 of the most intense ion signals were selected as precursors for MS/MS acquisition, excluding the trypsin autolysis peaks and the matrix ion signals. In MS/MS positive ion mode, for one main MS spectrum, 50 subspectra with 50 shots per subspectrum were accumulated using a random search pattern. Collision energy was 2 kV, the collision gas was air, and default calibration was set by using the Glu1-Fibrino-peptide B ((M + H) + 1570.6696) spotted onto Cal 7 positions of the MALDI target. Combined peptide mass fingerprinting PMF and MS/MS queries were performed by using the MASCOT search engine 2.2 (Matrix Science, London, UK) embedded into GPS-Explorer Software 3.6 (Applied Biosystems) on the NCBI Non-redundant Database with the following parameter settings: 100 ppm mass accuracy, trypsin cleavage with one missed cleavage allowed, carbamidomethylation set as a fixed modification, oxidation of methionine was allowed as variable modification, MS/MS fragment tolerance was set to 0.4 Da. A GPS-Explorer protein confidence index ≥95% were used for further manual validation.

### 4.6. Gene Ontology Analysis

Gene ontology (GO) analysis of differentially expressed proteins was performed by BLAST2GO software (version 2.7.2) [38]. To perform GO mapping and annotation, sequences of the differentially expressed proteins were inported into the software by setting the maximum number of blast hits to 20. The mapping step retrieved the GO terms associated with the blastx hits via the public BLAST2GO Database “b2g_sep13”. Then, the annotation step was carried out with default parameters to select reliable terms among those obtained in the mapping and to assign them to the queries. To summarize the functional classification of thedifferentially expressed proteins, resulting GO annotation was mapped to GO slim terms using the BLAST2GO internal mapping function using the “oslim_plant.obo” ontology subset.

### 4.7. RNA Isolation and qRT-PCR Assays

Total RNA from CYR32- or mock-inoculated rice leaves were sampled at 0, 12, 24, 48 and 72 hpi and extracted using the Trizol reagent (Life Technologies, Grand Island, NY, USA). DNase I (Fermentas, Shenzhen, China) treatment was applied to remove genomic DNA and first strand cDNA was synthesized with a RevertAid First Strand cDNA Synthesis Kit (Fermentas). Primers (see Supplementary Table S2) were specifically designed to anneal to each of the selected genes and the endogenous reference gene *OsActin* (Genbank accession No. KC140126). Expression patterns of selected genes were analyzed with a Bio-Rad iQ5 system. Relative gene quantification was calculated by the comparative 2^–ΔΔ*C*t^ method [39] and normalized to the corresponding expression level of the *OsActin*. All reactions were performed in triplicate, including three no-template controls.

## 5. Conclusions

In summary, we identified 27 differentially expressed proteins in rice in response to *Pst* inoculation using 2-DE and mass spectrometry. Most of these proteins are involved in basic resistance, such as glycerol-3-phosphate and hydrogen peroxide signaling. A homologue of wheat leaf rust resistance protein Lr10 was also identified, indicating that multiple defense layers are involved in rice NHR to *Pst* infection. These results demonstrate an intrinsic relationship between host and nonhost resistance and extend our knowledge on plant immune systems.

## Supplementary Materials

Supplementary materials can be found at http://www.mdpi.com/1422-0067/15/12/21644/s1.
